# Hand-Sewn Gastrojejunal Anastomosis in Laparoscopic Roux-en-Y Gastric Bypass: A Comparison of Absorbable Monofilament Versus Non-Absorbable Multifilament Sutures and Their Impact on the Symptomatic Anastomotic Stricture Rate

**DOI:** 10.3390/jpm16080431

**Published:** 2026-08-15

**Authors:** Hugo A. Sanchez, Guillermo Ponce de Leon-Ballesteros, Miguel F. Herrera

**Affiliations:** 1 The Nutrition & Obesity Clinic, ABC Medical Center, Sur 136 # 116, México City 01120, Mexico; miguelfherrera@gmail.com; 2 Department of Surgery, Hospital Angeles Morelia, Morelia 58350, Mexico; guillermoplb@hotmail.com; 3 Department of Surgery, Instituto Nacional de Ciencias Médicas y Nutrición Salvador Zubirán, Vasco de Quiroga 15, México City 14000, Mexico

**Keywords:** Roux-en-Y gastric bypass, hand-sewn anastomosis, anastomotic stricture, gastrojejunal anastomosis

## Abstract

**Introduction**: Gastrojejunal anastomosis (GJA) stricture is an important complication after laparoscopic Roux-en-Y gastric bypass (LRYGB). The impact of suture material on stricture rate when performing a hand-sewn GJA is not well established. **Methods**: This is a retrospective analysis of a prospectively maintained database that includes 882 patients who underwent primary or revisional LRYGB with a minimum 12-month follow-up. We compared two consecutive groups of patients who underwent hand-sewn gastrojejunal anastomosis: group 1 (*n* = 426), utilizing a multifilament non-absorbable/absorbable suture combination (silk/Vicryl^®^), whereas group 2 (*n* = 456) received an absorbable monofilament suture (Monocryl^®^). The primary outcome was the incidence of symptomatic GJA stricture, defined as an inability to pass a 10 mm gastroscope associated with dysphagia, vomiting, or food intolerance. **Results**: The symptomatic stricture rate was significantly lower in group 2 (0.7% vs. 6.8%; *p* < 0.001). On multivariate analysis, the use of monofilament sutures was a stronger protective factor (OR 0.09; 95% CI 0.03–0.30; *p* < 0.001), whereas revisional surgery was associated with an increased risk of symptomatic stricture (OR 2.61; 95% CI 1.05–6.50; *p* = 0.03). Type 2 diabetes mellitus and gender were not independent predictors. One-year weight loss was similar between patients who developed symptomatic strictures (and required dilations) and those who did not (*p* > 0.05). **Conclusion**: The use of absorbable monofilament sutures for hand-sewn GJA in LRYGB reduces the risk of symptomatic stricture without compromising weight loss outcomes. These findings support a personalized suture strategy, especially in high-risk patients undergoing revisional surgery.

## 1. Introduction

Laparoscopic Roux-en-Y gastric bypass (LRYGB) remains one of the most frequently performed bariatric procedures worldwide and is considered the longest-standing surgical option among currently utilized metabolic operations [1,2]. Since its introduction, the procedure has undergone numerous modifications and technical improvements aimed at optimizing metabolic results while reducing complications. These technical variations have included adjustments to the lengths of the biliopancreatic and alimentary limbs, the size of the gastric pouch, and, most critically, the method and material used to construct the gastrointestinal anastomosis [3,4,5]. Despite the relative standardization of many surgical steps, substantial heterogeneity persists in clinical practice, reflecting the absence of definitive evidence favoring a simple technical approach [1,6].

Among the components of LRYGB, construction of the gastrojejunal anastomosis (GJA) is widely regarded as the most technically demanding step [7,8]. This anastomosis is critical not only for achieving adequate restriction (a key mechanism of weight loss), but also because it represents a common source of postoperative morbidity. One of the most frequently described complications associated with this anastomosis is stricture or stenosis, which typically presents with progressive dysphagia, vomiting and food intolerance, followed by dehydration, nutritional deficiencies, and impaired quality of life [9,10,11,12]. The reported incidence of GJA stricture varies widely across series, ranging from 0.3% to 33%, a variability that reflects differences in surgical technique, definitions used for diagnosis, and patient selection [12,13,14,15,16,17,18,19,20,21,22].

The pathophysiology of GJA strictures is multifactorial. Late-onset strictures (occurring months to years after surgery) have been associated with patient-related factors such as type 2 diabetes mellitus, advanced age, smoking, and chronic use of non-steroidal anti-inflammatory drugs (NSAIDs) [23,24]. Conversely, early strictures, typically presenting within the initial 2–3 postoperative months, are predominantly attributable to technical factors related to anastomotic construction, including ischemia, excessive tension, submucosal hematoma, subclinical leaks, or the inherent characteristics of the suture or stapler device used [12,19,21,22]. The timely identification of these technical determinants may enable preventive strategies and reduce the incidence of this complication.

Several techniques for constructing GJA have been described, including circular and linear stapled, and completely hand-sewn anastomoses. A general consensus in the literature suggests that the incidence of stricture varies by the technique, with the highest stricture rates occurring with circular stapled anastomoses, particularly when a 21 mm stapler is employed [21,23], whereas linear stapled and hand-sewn techniques generally yield lower rates [9,13,15,16,20,24,25,26,27]. A systematic review by Jiang et al. demonstrated that hand-sewn anastomosis is associated with a trend toward lower stricture rates compared to mechanical techniques, although the difference did not always reach statistical significance [8]. Relatively few studies have specifically examined the impact of different suture materials on stricture formation when a hand-sewn technique is consistently applied [28,29]; among these, one prospective cohort study reported an important reduction in stricture incidence from 9.5% with multifilament absorbable sutures to 0.7% with monofilament sutures, suggesting that suture selection may significantly influence outcomes [30]. This finding identifies the potential importance of the inflammatory response and tissue reaction elicited by different suture types in the healing process of the GJA. Personalized surgical strategies require identifying modifiable technical factors, such as the suture material used, that can be tailored to individual patient risk profiles.

Given the scarcity of comparative data on suture materials in hand-sewn GJA and the considerable variability in reported stricture rates, this study aims to evaluate and compare the incidence of gastrojejunal symptomatic anastomotic stricture following primary LRYGB by using two different suture materials: a non-absorbable/absorbable, multifilament suture combination (silk/polyglactin 910) versus an absorbable, monofilament suture (polyglecaprone 25). The primary objective is to determine if the choice of suture material affects the rate of anastomotic stenosis. Secondary objectives include assessing the impact on weight loss outcomes and the need for subsequent endoscopic or surgical interventions.

## 2. Materials and Methods

### 2.1. Study Design and Settings

This was a retrospective analysis of a prospectively maintained database of consecutive patients who underwent primary or revisional LRYGB at the Centro Médico ABC Observatorio, México City, between March 2004 and April 2024. All procedures were performed by a single surgical team composed of two bariatric surgeons who developed the same standardized surgical technique, and the study was approved by the institutional review board (IRB No. CMABC-25-50). Owing to the retrospective nature and the use of de-identified data, the requirement for informed consent was waived.

During the early period of our experience (2004–2010), we identified a high incidence of gastrojejunal anastomotic stricture during postoperative follow-up, and this prompted a change in our clinical practice. We switched from a combination of multifilament sutures to absorbable monofilament sutures, as described below. All other components of the surgical procedure remained unchanged.

All patients undergoing LRYGB during the study period were eligible for inclusion. The procedure was performed according to the international bariatric surgery guidelines applicable at the time of each intervention, and a complete preoperative work-up, including medical, nutritional, and psychological evaluations, was carried out. A minimum follow-up of 12 months with at least one documented clinical assessment was required, including weight loss and the status of comorbid conditions. Exclusion criteria included chronic use of NSAIDs or corticosteroids, and anastomotic leaks.

### 2.2. Surgical Technique

All operations were performed using five ports. A 30 mL gastric pouch was constructed along the lesser curvature of the stomach using linear staplers, and gastrointestinal continuity was restored in a Roux-en-Y fashion. The alimentary limb was measured to approximately 100 cm, and the biliopancreatic limb between 75 and 100 cm. The jejunojejunostomy was created side-to-side using a 60 mm linear stapler. The gastrojejunostomy was hand-sewn in two planes over a 30-French bougie, which gives a diameter close to 2 cm, using four continuous suture lines in an antecolic and antegastric fashion. The Petersen space and the mesenteric defect at the jejunojejunostomy were routinely closed with non-absorbable sutures. As previously described, patients were divided into 2 groups according to the suture material used for the hand-sewn gastrojejunostomy. In group 1 (from March 2004 to October 2010), a multifilament suture combination was used: 3-0 silk (non-absorbable, multifilament) for external layers and 3-0 Vicryl^®^ (Polyglactin 910, absorbable, multifilament; Ethicon, Somerville, NJ, USA) for the internal layers. In group 2 (from November 2010 to April 2024), an absorbable monofilament suture, 3-0 Monocryl^®^ (Polyglecaprone 25; Ethicon, Somerville, NJ, USA), was used for all four suture lines.

### 2.3. Study Variables

The primary outcome was the incidence of symptomatic stricture of the gastrojejunostomy, defined as the inability to pass a 10 mm gastroscope through the anastomosis in the presence of dysphagia, vomiting, and food intolerance. The main independent variables were the type of suture strategy (monofilament absorbable vs. multifilament combination) and the type of surgery (primary vs. conversion). Additional variables included age, gender, body mass index (BMI), operative time, type 2 diabetes mellitus, percentage of excess weight loss (%EWL), and percentage of total weight loss (%TWL) at 12 months.

### 2.4. Statistical Analysis

Statistical analysis was performed using IBM SPSS Statistics version 26 (IBM Corp., Armonk, NY, USA) and Microsoft Excel for MAC version 16.46. We expressed continuous variables as means ± standard deviations (range) or as the median with an interquartile range, depending on the distribution. Categorical variables are shown as absolute frequencies and percentages. For comparisons between the two groups, we used Student’s *t*-test or the Mann–Whitney U test for continuous variables, and the chi-square (χ^2^) test or Fisher’s exact test was used for categorical variables.

To assess the independent impact of suture type on stricture incidence, we first performed a bivariate analysis of clinically relevant risk factors, followed by a binary logistic regression model adjusted for the type of surgery (primary vs. conversion) and diabetes mellitus. The results are expressed as odds ratios (OR) with 95% confidence intervals (CI). A *p*-value < 0.05 was considered statistically significant. Neither smoking status nor endoscopic preoperative status (EPS) was included as a covariate due to inconsistent and non-systematic capture in our database. To assess for a potential learning curve effect over time, we performed a simple linear regression using the calendar year as the predictor and logit of the stricture rate as the outcome.

## 3. Results

A total of 1072 patients underwent LRYGB during the study period (502 in group 1 and 570 in group 2). Of these, 882 patients (82.2%) completed the minimum 12-month follow-up and constituted the study group (426 in group 1 and 456 in group 2). Baseline demographic and clinical characteristics of the two groups are summarized in Table 1. The groups were comparable with respect to gender, initial BMI, and operative time. Patients in group 2 were older and had a higher prevalence of type 2 diabetes mellitus. The proportion of revisional procedures (conversion from gastric banding or sleeve gastrectomy to LRYGB) was 8.9% and 12.5% in groups 1 and 2, respectively (*p* = 0.08).

A symptomatic stricture of the gastrojejunostomy was found in 29 patients (6.8%) of group 1, which is significantly higher than the 3 patients (0.7%) who developed the same complication in group 2 (*p* < 0.001) (Table 2).

In a subgroup analysis according to the suture type and type of surgery, the protective effect of a monofilament absorbable suture was most pronounced in primary procedures. The stricture rate was lower in primary procedures (3.2%) than in revisional procedures (7.4%), although this difference did not reach statistical significance (*p* = 0.07) (Table 3).

In the multivariate analysis, the use of monofilament absorbable suture was the strongest protective factor, reducing the risk of stricture by more than 90% (OR 0.09, 95% CI 0.03–0.30, *p* < 0.001). This finding identifies an opportunity for personalized application of this strategy, particularly in high-risk subgroups, such as those undergoing revisional surgery, which was associated with a 2.6-fold increased risk of stricture (OR 2.61, 95% CI 1.05–6.50, *p* = 0.03). Diabetes mellitus was not an independent predictor in the multivariate model (OR 0.41, 95% CI 0.12–1.37, *p* = 0.15). Male gender showed a trend toward lower risk but did not reach statistical significance (OR 0.58, 95% CI 0.24–1.41, *p* = 0.23) (Table 4). We also checked whether the year of surgery influenced the risk of stricture. The regression showed no significant trend *(β* = −0.141; 95% CI for the slope: −0.436 to 0.154; *p* = 0.273). This translates to an annual odds ratio of 0.87 (95% CI: 0.65–1.17), meaning that the small year-to-year variation was not statistically significant. Therefore, we did not include “year” in the multivariate model to avoid statistical overfitting, given the limited number of events (n = 32).

Weight loss was compared between patients who developed symptomatic stricture and therefore required endoscopic dilation (n = 32), and those who did not (n = 850). No significant differences were observed at 1 year in BMI, %EWL, or %TWL (Table 5), indicating that the presence of a stricture/dilation did not adversely affect weight loss.

## 4. Discussion

Several factors have been associated with optimal weight loss, among which the diameter of the gastrojejunostomy has been identified as one important determinant [31,32,33]. Numerous modifications in materials and techniques have been attempted to achieve a smaller stoma while minimizing associated adverse effects, such as leaks and strictures. The optimal GJA diameter likely lies between 15 and 30 mm, a range that provides sufficient restriction for bariatric purposes [31]. In our study, we were able to demonstrate a very low stricture rate of hand-sewn gastrojejunostomies close to 2 cm in diameter using absorbable monofilament sutures and a significant reduction of strictures when compared with patients in whom a combination of multifilament sutures (silk and Vicryl^®^) was used.

The incidence of gastrojejunal stricture after LRYGB varies widely in the literature, ranging from 0.03% to 27%, depending on the anastomotic technique, stapler diameter, suture material, and the definition used [9,17,21]. For hand-sewn anastomosis, reported stricture rates are generally lower than those for circular stapled techniques [25,27] but still range from 3% to 10% in many series [16,34]. Our stricture rate of 6.8% with a multifilament suture is consistent with these reports, whereas the rate of 0.7% with a monofilament suture is among the lowest reported for any hand-sewn technique.

The prospective study by Ruiz de Adana et al. is of extraordinary value for comparison, as it first reported a decrease in the stricture rate from 9.5% with a multifilament absorbable suture (Vicryl^®^) to 0.7% with a monofilament absorbable suture (Monocryl^®^) in a single-surgeon series [30]. Our results closely mirror these findings despite differences in patient populations since our patients had a lower mean BMI and we included revisional procedures. The consistency of this observation across two independent institutions and different time periods strongly supports the conclusion that the monofilament absorbable suture is superior to the multifilament suture for preventing stricture in a hand-sewn gastrojejunostomy.

We acknowledge that the comparison of monofilament versus multifilament sutures might be considered a historical discussion in other fields of gastrointestinal surgery in which the impact of suture material on the anastomotic diameter is less evident, likely because in these procedures the anastomosis is intentionally constructed as wide as possible to minimize the risk of postoperative stenosis [35,36,37]. By contrast, in bariatric surgery, the gastrojejunal anastomosis is deliberately constructed with a small diameter to achieve the desired restrictive effect [31,32,33]. Consequently, any factor that induces even a modest degree of excessive inflammation or fibrosis (such as the use of multifilament braided sutures) can convert an already narrow stoma into a clinically symptomatic stricture. This explains why the impact of suture material on stricture rate is uniquely evident in bariatric surgery, whereas this effect may be less apparent in other gastrointestinal anastomoses.

The mechanism underlying this difference is likely related to the physical properties and tissue reaction of the suture materials. Multifilament braided sutures theoretically have a higher coefficient of friction, may carry and host bacteria, and induce a more pronounced inflammatory response, which can lead to excessive fibrosis and scarring at the anastomotic site [30]. In contrast, monofilament sutures are smoother, less thrombogenic, and may induce a milder inflammatory reaction, allowing for more physiological tissue repair. The absorbable nature of Monocryl^®^ (complete hydrolysis by 90–120 days) also eliminates any long-term foreign body reaction, whereas permanent or slowly absorbable multifilament sutures may perpetuate chronic inflammation and promote stricture formation.

A methodological consideration is that our two suture strategies differed in both structure and absorbability. Nevertheless, rather than merely highlighting the adverse effects of the non-absorbable component, our findings primarily underscore the remarkably positive performance of the absorbable monofilament strategy, which yielded a stricture rate of only 0.7%, a figure that compares favorably with the lowest rates reported in the literature for hand-sewn gastrojejunostomies, including the 0.7% benchmark reported by Ruiz de Adana et al. [30]. This suggests that complete suture absorption eliminates chronic foreign body inflammation, allowing for more physiological tissue remodeling and optimal anastomotic healing. Therefore, our data support adopting a personalized strategy that combines monofilament configuration with full absorbability, particularly for high-risk patients such as those undergoing revisional surgery. Future randomized controlled trials comparing all four suture combinations (absorbable vs. non-absorbable, and multifilament vs. monofilament) would be valuable to isolate the independent contributions of filament structure and absorbability.

In recent years, barbed (“or knotless”) monofilament absorbable sutures have been incorporated into hand-sewn anastomosis techniques. Practical advantages are to eliminate the need for knot-tying and to maintain constant tension without assistance, which may reduce operative time and technical difficulty. A systematic review and meta-analysis by Ataya et al. (2024) confirmed that barbed sutures significantly reduce the suturing time (mean difference −4.87 min; 95% CI −8.43 to −1.30; *p* < 0.01) and overall operative time in LRYGB (mean difference −12.11 min; 95% CI −19.27 to −4.95; *p* < 0.01), with no significant differences in postoperative complications such as leak, bleeding, stenosis, or bowel obstruction [28]. Similarly, the meta-analysis by Chaouch et al. (2021) reported that barbed sutures are associated with a shorter operative time (MD -7.90 min; 95% CI −12.95 to −2.84; *p* = 0.002) and similar rates of anastomotic leak (OR 1.25), stricture (OR 0.89), and bleeding (OR 0.62) compared to conventional multifilament sutures [38]. A large propensity-score matched study by Pennestrì et al. (2019) also concluded that barbed sutures are effective for closing the gastric pouch-jejunal anastomosis and are safe as conventional sutures, with significantly shorter operative times in the LRYGB group (*p* < 0.001) [39].

However, while we acknowledge that barbed sutures offer practical advantages in terms of ease and speed and are not associated with an increased risk of anastomotic complications [28,29,38,39,40], we favored non-barbed monofilament absorbable sutures due to theoretical concerns about tissue trauma from the barbs, which are designed to engage the tissue and prevent back-sliding but create multiple points of contact and micro-trauma along the suture track. We also acknowledge that the use of a non-barbed monofilament absorbable suture is technically more demanding than the use of barbed or braided sutures.

Although the absolute number of conversion cases in our series was modest (n = 95), the 7.4% symptomatic stricture rate in this subgroup identified revisional bariatric surgery as an independent predictor of anastomotic stricture (OR 2.61, *p* = 0.03), probably due to a more technically demanding procedure resulting from adhesions, scar tissue, and altered anatomy, which may compromise the blood supply to the anastomosis or increase tension on the suture line [12]. Also, patients undergoing revisional surgery tend to have a longer history of obesity and more complex metabolic profiles, emphasizing the need for increased vigilance and possibly additional intraoperative precautions, such as atraumatic handling of tissues and ensuring a tension-free anastomosis.

Weight loss after dilation of the gastrojejunostomy might be a concern. But our results show that patients with symptomatic strictures had no difference in weight loss from those without. This finding is consistent with previous reports stating that a successful endoscopic dilation of a stenosed gastrojejunostomy restores adequate caloric intake without compromising the restrictive effect of the gastric bypass [21,34].

All 32 patients with symptomatic strictures in our series were successfully treated with endoscopic balloon dilation. No patient required surgical revision for refractory stricture, and no major complications (perforation, severe bleeding) occurred. This confirms that endoscopic dilation is a safe and effective first-line treatment for post-RYGB anastomotic strictures, as reported by several large series [10,22].

Several limitations must be acknowledged. This was a retrospective, non-randomized study with two consecutive chronological cohorts. Although the surgical technique remained unchanged except for the suture material, the 20-year study period introduces a potential learning curve effect, as improvements in laparoscopic instrumentation, surgical experience, and perioperative protocols may have reduced morbidity in later years. To address this, we formally analyzed the stricture rate by calendar year. The regression showed no significant temporal trend (*p* = 0.27), suggesting that surgical experience over time did not substantially alter the stricture rate. This is consistent with the abrupt drop-in stricture rate from 6.8% to 0.7% immediately after switching sutures.

Furthermore, routine postoperative endoscopy was not performed in asymptomatic patients. As a result, our stricture rate reflects only clinically apparent cases, and the true overall incidence of anastomotic strictures may be higher than reported. For this reason, our findings and conclusions should be interpreted strictly in the setting of symptomatic anastomotic stenosis.

Finally, as acknowledged in the Section 2.4, smoking status and *H. pylori* status were not systematically captured in our database and were therefore not included as covariates. Both are well-established risk factors for marginal ulceration and subsequent anastomotic stricture [9,24]. Their omission introduces potential unmeasured confounding, which limits the robustness of our multivariate logistic regression model. Although our model adjusted for the main surgical and demographic variables, we cannot exclude residual confounding from these factors, and our results should be interpreted with this limitation in mind.

This study has several strengths, including a large sample size (882 patients) with a complete 12-month follow-up, the homogeneity of the surgical technique (single team, same operative steps except for the suture material), and the prospective nature of our database. The multivariate analysis, adjusted for important confounders such as conversion surgery and diabetes, which are well-established risk factors for anastomotic stricture in the literature [9,12,24], showed that the protective effect of monofilament sutures persisted across these high-risk subgroups. This reinforces the conclusion that the suture material itself is an important determinant of stricture formation.

## 5. Conclusions

The use of absorbable monofilament sutures for a hand-sewn gastrojejunostomy in LRYGB significantly reduces the risk of symptomatic strictures compared with a multifilament non-absorbable/absorbable combination. These findings support a personalized suture strategy, especially in high-risk patients undergoing revisional surgery.

## Figures and Tables

**Table 1:** Baseline characteristics of the studied groups.

Variable	Group 1 (n = 426)	Group 2 (n = 456)	*p*-Value
Gender (male/female), n	174/252	172/284	0.34 *
Conversion surgery, n (%)	38 (8.9)	57 (12.5)	0.08 *
Age at surgery (years), median (IQR)	39 (31–47)	42 (35–50)	<0.001 **
Initial weight (kg), median (IQR)	116 (104.7–135.4)	114.4 (99.6–134.3)	0.03 **
Initial BMI (kg/m^2^), median (IQR)	41.7 (38.1–46.4)	41.4 (37.6–45.6)	0.21 **
Operative time (min), median (IQR)	120 (120–180)	150 (120–180)	0.06 **
Type 2 diabetes mellitus, n (%)	87 (20.4)	155 (34.0)	<0.001 *

IQR, Interquartile range; * χ^2^ test; ** Mann–Whitney U test.

**Table 2:** Bivariate analysis of risk factors for gastrojejunal symptomatic anastomotic stricture.

Variable	Stenosis n (%)	No Stenosis n (%)	*p*-Value *
**Suture strategy**			
Multifilament combination (silk + Vicryl^®^)	29 (6.8)	397 (93.2)	<0.001
Monofilament absorbable (Monocryl^®^)	3 (0.7)	453 (99.3)	
**Type 2 diabetes**			
Yes	3 (1.2)	239 (98.8)	0.02
No	29 (4.5)	611 (95.5)	
**Gender**			
Male	7 (2.0)	339 (98.0)	0.04
Female	25 (4.7)	511 (95.3)	

Pearson’s chi-squared test. * Pearson’s chi-squared test.

**Table 3:** Stenosis rate according to type of surgery and suture.

Procedure Type	Suture Strategy	Stenosis n (%)	No Stenosis n (%)	*p*-Value (Suture Comparison)	OR (95% CI)
**Primary (n = 787)**		25 (3.2)	762 (96.8)		
	Multifilament combination	24 (6.2)	364 (93.8)	<0.001	26.25 (3.54–195.6)
	Monofilament absorbable	1 (0.3)	398 (99.7)		
**Revisional (n = 95)**		7 (7.4)	88 (92.6)		
	Multifilament combination	5 (13.2)	33 (86.8)	0.11	4.17 (0.77–22.8)
	Monofilament absorbable	2 (3.5)	55 (96.5)		

Global comparison vs. revisional (regardless of suture type): *p* = 0.07 (Fisher’s exact test), OR 2.42 (95% CI 1.02–5.76).

**Table 4:** Binary logistic regression analysis for risk of symptomatic stricture of the gastrojejunostomy.

Variable	Category	OR (95% CI)	*p*-Value
Suture strategy	Monofilament absorbable vs. multifilament combination	0.09 (0.03–0.30)	<0.001
Type of surgery	Conversion vs. primary	2.61 (1.05–6.50)	0.03
Diabetes mellitus	Yes vs. no	0.41 (0.12–1.37)	0.15
Gender	Male vs. Female	0.58 (0.24–1.41)	0.23

Model summary: Omnibus test *p* < 0.001; Nagelkerke R^2^ = 0.16.

**Table 5:** Weight loss at 12 months according to stricture status.

Variable	Stricture/Dilation Group (n = 32)	No Stricture/Dilation Group (n = 850)	*p*-Value
BMI (kg/m^2^)	28.5 ± 5.3	28.7 ± 4.9	0.79 *
Excess weight loss (%)	76.9 ± 28.5	77.9 ± 21.9	0.79 *
Total weight loss (%)	30.2 ± 11.1	32.3 ± 8.4	0.17 *

All values are mean ± SD. Student’s *t*-test.

## Data Availability

The datasets generated and/or analyzed during the current study are not publicly available due to privacy and ethical restrictions, as they contain potentially identifying patient information. De-identified data may be made available by the corresponding author upon reasonable written request, subject to institutional review board approval (IRB No. CMACB-25-50) and the execution of a data access agreement to ensure confidentiality.
